# The Multifaceted Role of Long Non-Coding RNA in Gastric Cancer: Current Status and Future Perspectives

**DOI:** 10.7150/ijbs.61410

**Published:** 2021-06-26

**Authors:** Yifan Li, Lan Lu, Xu Wu, Qianxiu Li, Yueshui Zhao, Fukuan Du, Yu Chen, Jing Shen, Zhangang Xiao, Zhigui Wu, Wei Hu, Chi Hin Cho, Mingxing Li

**Affiliations:** 1 Laboratory of Molecular Pharmacology, Department of Pharmacology, School of Pharmacy, Southwest Medical University, Luzhou 646000, Sichuan, China.; 2 South Sichuan Institute of Translational Medicine, Luzhou 646000, Sichuan, China.; 3 Antibiotics Research and Re-evaluation Key Laboratory of Sichuan Province,Sichuan Industrial Institute of Antibiotics, School of Pharmacy, Chengdu University, Chengdu 610106, Sichuan, China.; 4 Department of Pharmacy, the Affiliated Hospital of Southwest Medical University, Luzhou 646000, Sichuan, China.; 5 Department of Gastroenterology, Shenzhen Hospital, Southern Medical University, Shenzhen 518000, Guangzhou, China.

**Keywords:** long non-coding RNA, gastric cancer, drug resistance, *helicobacter pylori*, tumor microenvironment

## Abstract

Gastric cancer (GC) is one of the major public health concerns. Long non-coding RNAs (lncRNAs) have been increasingly demonstrated to possess a strong correlation with GC and play a critical role in GC occurrence, progression, metastasis and drug resistance. Many studies have shed light on the understanding of the underlying mechanisms of lncRNAs in GC. In this review, we summarized the updated research about lncRNAs in GC, focusing on their roles in *Helicobacter pylori* infection, GC metastasis, tumor microenvironment regulation, drug resistance and associated signaling pathways. LncRNAs may serve as novel biomarkers for diagnosis and prognosis of GC and potential therapeutic targets. The research gaps and future directions were also discussed.

## Introduction

Gastric cancer (GC) is among the most common human malignancies and is the third leading cause of cancer death worldwide 1. Approximately one million new cases are diagnosed each year. Although great advances have been gained in early diagnosis and management of GC 2, it still remains a major public health problem. GC is caused by the interaction of host genetic factors and complex environmental factors and manifested by complex cross talks within tumor microenvironment. *Helicobacter pylori* (*H. pylori*) infection has been suggested as one of the most important drivers in GC tumorigenesis 3. Most GC patients can prolong their overall survival and improve life quality through the combinational use of chemotherapeutics and targeted therapy. However, the multi-drug resistance (MDR) of GC is a major restrain in clinical practice.

It is revealed that protein-coding RNAs account for less than 2% of the total transcripts, while the rest of them are non-coding RNAs (ncRNAs) that lack the ability to code proteins. However, the increasing evidence shows that ncRNAs play a crucial role in different cellular processes from normal development to disease process 4. The ncRNAs are separated into two major types based on their size: (1) small ncRNAs with a molecular length of no longer than 200 base pairs (bps), represented by microRNAs and transfer RNAs; (2) long ncRNAs (lncRNAs) with more than 200 bps in length. In the past decades, for small ncRNAs, especially the microRNAs (miRNAs), their biological functions have been extensively explored. On the other hand, the underlying knowledge about the functional role of lncRNAs is rather limited, as they were often considered to be transcription noises. Owing to the advantages of next-generation sequencing technologies, lncRNAs have received extensive attention in different areas. Emerging evidence indicates that lncRNAs play a vital role in regulating various cellular processes through several ways including sponging miRNAs, interaction with proteins and modulation of target gene expressions 5. Specifically, given the multifaceted roles of lncRNAs in GC tumorigenesis and progression, in this work, we will comprehensively review the function of lncRNAs in GC development and discuss their interactions with GC tumor microenvironment and the impact on GC drug resistance.

## LncRNAs and *H. pylori* Infection

*H. pylori* infection exhibits a pivotal role in gastric tumorigenesis, which is infected by more than 70% of all patients 3. During the past years, numerous studies have demonstrated that the crucial role of protein-coding genes and genomic variations in the pathogenesis of GC. It is increasingly realized that lncRNAs, which fail to code protein, may become a big part of the occurrence and progression of various diseases. It has become apparent that lncRNAs are associated with GC induced by *H. pylori* infection. In this work, we summarized the interactions between lncRNAs and *H. pylori*-associated GC.

### Association of the Expression Level of LncRNAs with *H. pylori*-Related GC

Some lncRNAs play an oncogenic role in GC. A recent study showed that lncRNA H19 was significantly upregulated in tumors of GC patients with *H. pylori* infection 6, 7. Overexpression of lncRNA H19 was correlated with all inflammatory indexes and promoted proliferation, migration and invasion in GC cells infected by *H. pylori*. Notably, Receiver operating characteristic (ROC) analysis aiming at exploring the potential markers for distinguishing stages III to Ⅳ of GC showed that lncRNA H19 preformed high diagnostic accuracy (95.5%), specificity (100%) and sensitivity (90.9%) 6, 7. This study suggested that lncRNA H19 could be a diagnostic marker for *H. pylori*-associated GC. Jin *et al.* found a significant increase of lncRNA HULC in the serum of GC patients, and overexpressed lncRNA HULC was significantly correlated with tumor growth and metastasis, as well as *H. pylori* infection 8. In Jing *et al.*'s study 9, they found that the expression of lncRNA PVT1 was remarkably elevated in normal gastric epithelial cells GES-1 which were infected by *H. pylori*. Intriguingly, the inflammatory markers caused by *H. pylori* infection were inhibited upon knockdown of lncRNA PVT1. The result suggested that lncRNA PVT1 may function as a pro-inflammatory factor and led to the tumorigenesis of *H. pylori*-associated GC 9. Moreover, it was found that the aberrant expression of GC-associated lncRNA 1 (GClnc1) contributed to pathological differentiation, vascular invasion, tumor size and poor prognosis in GC 10. GClnc1 was increasingly expressed from normal stomach tissue to intestinal metaplasia (IM), to dysplasia, and to GC, suggesting that overexpressed GClnc1 may be a diagnostic index for early stage of GC. Mechanistically, GClnc1 could interact with the complex of WDR5 (a key component of histone methyltransferase complex) and KAT2A histone acetyltransferase, leading to an alteration of the target gene histone modification pattern and thus enhancing the progression of GC. Similarly, overexpression of GClnc1 was also observed in GC tissues with* H. pylori* infection. However, the accurate role of GClnc1 on *H. pylori*-induced GC development was indistinct 10.

On the contrary, evidence also suggests that lncRNAs function as tumor suppressor, which had been implicated in the development of GC following *H. pylori* infection. For example, lncRNA AF147447 was able to suppress the expression of MUC2 through interacting with miRNA-34c, and the lncRNA AF147447 promoter could recruit the transcription factor E2F1 11. This suggested that lncRNA AF147447 was involved in GC development acting as a tumor suppressor, which was inhibited by *H. pylori* infection. In addition, lncRNA NR_026827 was found downregulated in GES-1 cells infected by* H. pylori*, and had a lower expression in GC tissues, comparing with normal tissues. However, the exact role of lncRNA NR_026827 in *H. pylori*-associated GC remains inconclusive 12.

Many studies have investigated the profiles of lncRNAs in *H. pylori*-infected cells and tumors. By using microarray analysis, Yang *et al.* identified some lncRNAs which were aberrantly expressed in *H. pylori*-infected gastric epithelial cells 13. They found that expressions of 23 lncRNAs were upregulated, while 21 lncRNAs were downregulated. Further analysis revealed that, among the aforementioned 44 lncRNAs, only the expression level of XLOC-004122 and XLOC-014388 was decreased in the gastric mucosal samples of patients infected by *H. pylori* 13. Another study by Yang *et al.* also recognized that some lncRNAs were abnormally regulated in *H. pylori*-associated GC, which may have prognostic values 14. Notably, RP11-169F17.1 and RP11-669N7.2 were found significantly correlated with poor overall survival and were remarkably related with *H. pylori* infection 14. Moreover, in Yang* et al.*'s earlier investigations, 765 *H. pylori* infection-associated genes were obtained based on miRNA-mRNA interaction network 15. Using Venn diagram, they indicated that 41% of RP11-169F17.1-related targets and 40.9% of RP11-669N7.2-related targets were overlapped with genes associated with *H. pylori* infection, respectively. There were an obvious positive association between the expression levels of two mentioned lncRNAs and their target genes. Additionally, these target genes were closely associated with GC 15. Taken together, the results suggest that RP11-169F17.1 and RP11-669N7.2 lncRNAs actively participate in the process of *H. pylori*-promoted gastrointestinal diseases including GC. It would be more convincing if the correlation between RP11-169F17/RP11-669N7.2 lncRNAs and *H. pylori*-associated gastric diseases is further clarified in animal models.

Additionally, it was found that 303 lncRNAs as well as 565 mRNAs were recognized as aberrantly expressed RNAs (*p* < 0.05) in the cells infected by *H. pylori* comparing with control 16. Quantitative real time polymerase chain reaction (qRT-PCR) analysis verified the expression profile of 8 lncRNAs in *H. pylori*-infected cells, including n345630, XLOC_004787, LINC00152, n378726, XLOC_005517, LINC00473, XLOC_13370 and n408024. Four lncRNAs were found down-regulated in the cells infected by *H. pylori*, which may be significantly correlated with the pathological effects of *H. pylori* 16. Nonetheless, the precise mechanisms and downstream targets are required to be explored in future studies.

In conclusion, many lncRNAs are found abnormally expressed in *H. pylori*-associated GC, some of which may have diagnostic or prognostic values. Nevertheless, in most cases, *in vitro* and *in vivo* experiments should be conducted in future investigations to confirm the exact roles and mechanisms of these lncRNAs in *H. pylori*-infected GC.

### Regulatory Networks of LncRNAs in *H. pylori*-Associated GC

It has been suggested that lncRNAs participated in regulation of *H. pylori*-associated GC by targeting specific genes via different signaling pathways. Glucocorticoid-inducible kinase 1 (SGK1) is an effective stimulator of multiple ion channels, which is capable of regulating cell proliferation, migration, cell volume, exocytosis, excitation and epithelial transport, and thus promotes tumor growth 17, 18. Interestingly, Yao *et al.* found that in intertumoral or peripheral T lymphocytes an increase in expression levels of SGK1 and its upstream lncRNA named lnc-SGK1 were observed in *H. pylori*-associated gastric carcinoma, which had a strong association with *H. pylori* infection and a high-salt diet (HSD). Moreover, lnc-SGK1 increased the transcription of SGK1 in a cis-regulatory way, and lnc-SGK1 may increase T helper 2 (Th2) and Th17 while decrease the differentiation of Th1 through SGK1/JUNB pathway. An elevated expression of lnc-SGK1 in serum upon *H. pylori* infection and/or HSD and in T lymphocytes was related to poor prognosis of GC patients, thus indicated that it may be an ideal diagnostic marker in human GC 18.

In another study, the lnc-GNAT1-1 was dramatically downregulated in GC cells infected by *H. pylori*, comparing to normal cells. *H. pylori* infection could suppress the expression level of lnc-GNAT1-1 in tumor xenografts. Overexpression of lnc-GNAT1-1 inhibited cell proliferation, migration and invasion in SGC-7901 and MKN45 cells via suppression of Wnt/β‑catenin signaling 19. These data revealed that lnc-GNAT1-1 played a role in regulating Wnt/β‑catenin pathway in *H. pylori*-associated GC.

**Figure 1:**
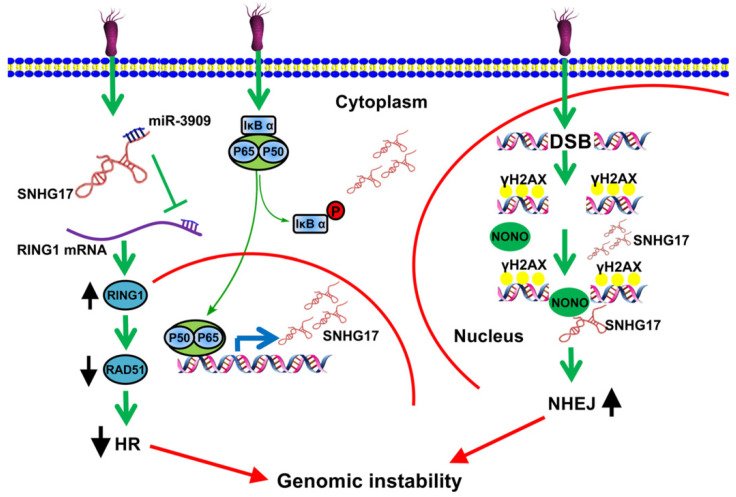
LncRNA SNHG17 participated in deregulation of NONO and miRNA-3909/RING1/Rad51 pathway during *H. pylori* infection and promoted the cells more prone to genetic rearrangements, therefore enhancing tumorigenesis in GC 25. Reprinted with permission 25.

Numerous studies have revealed that *H. pylori* infection causes DNA damage and influences DNA repair 20-23. DNA damage can lead to DNA double-strand breaks (DSBs) 24. To deal with these physical damages, there are two major repairing patterns in eukaryotic cells: one is through homologous recombination (HR), which utilizes homologous DNA sequences as templates and guarantees error-free repairing; another is through nonhomologous end joining (NHEJ), which recruits DNA-dependent protein kinase catalytic submit (DNA-Pkcs) to DNA break ends upon lacking the homologous recombination. Of great interest, Han *et al.* found that lncRNA SNHG17 was elevated due to *H. pylori* infection and highly-expressed lncRNA SNHG17 was correlated with low survival rate of GC patients 25. Notably, up-regulated nuclear lncRNA SNHG17 caused the recruitment of NONO, which together functioned as an abduction of miRNA-3909, modulating Rad51 expression level and thus resulting in an alteration of DSBs repairing pathways from HR to NHEJ. These findings indicated that lncRNA SNHG17/miRNA-3909/Rad51 pathway may contributed to progression of GC induced by *H. pylori* through affecting the DNA repair pathway (Fig. 1) 25.

Based on current findings, although some studies identified some novel lncRNAs which were aberrantly expressed in *H. pylori*-associated GC and paid attention to the regulatory network of lncRNAs, most of the downstream targets and action mechanisms were still mysterious. There still remain questions. For example, cytotoxin associated gene A (cagA) and vacuolating cytotoxin A (vacA) are two of the most important pathogenic factors in *H. pylori*, few studies had addressed the possible interactions between lncRNAs and these pathogenic markers. Thus, the potential contribution of dysregulated lncRNAs in multiple pathogenic factors induced by *H. pylori* needs more investigations.

In summary, the current findings extend our understanding in the crucial roles of dysregulation of lncRNAs and open novel investigation lines toward the potential lncRNA-related target for treatment or diagnosis of *H. pylori*-associated GC.

## LncRNAs and Metastatic GC

Cancer metastasis is one of the main features of GC due to late diagnosis, leading to an acceleration of malignant progression. Many lncRNAs, as one of key drivers, have been demonstrated to promote GC metastasis. A recent study showed that the expression of lncRNA AC093818.1 was positively correlated with invasion, lymphatic metastasis, distal metastasis, and tumor-node-metastasis stage of GC, which promoted GC metastasis through epigenetically inducing PDK1 expression both* in vitro* and* in vivo* 26. On the other hand, Zhang* et al.* found that lncRNA DRAIC could suppress GC metastasis of GC cells via through influencing NFRKB de-ubiquitination induced by UCHL5 27.

EMT is considered to be a core factor of tumor metastasis, and it is clear that a variety of lncRNAs participated in GC development by regulating this cell program. For instance, Li *et al.* observed that lncRNA MAG12-AS3 was overexpressed in GC tissues based on bioinformatics analysis, and high-expressed MAG12-AS3 was positively correlated with poor prognosis 28. Mechanically, further studies indicated that MAG12-AS3 was identified to an EMT-related lncRNA as it could significantly regulate ZEB1 (Zincfinger ebox binding homeobox 1) expression, while it sponged miRNA-141/200a and suppressed their expressions in GC 28. Moreover, it was confirmed that the expression level of lncRNA DLX6-AS1 was remarkably elevated in GC cells, and high-expressed DLX6-AS1 could enhance cell proliferation, migration and EMT process of GC cells 29. MAP4K1 (Mitogen-activated protein kinase 1) was positively regulated by lncRNA DLX6-AS1, and this biological process depended on the mediation of Fused sarcoma translocated in liposarcoma (FUS) 29.

Evidence has shown that lncRNAs have played an essential role in GC metastasis. However, till now it remains largely elusive that to what extent lncRNAs participate in promoting or inhibiting GC metastasis as well as whether and how they cooperate with other factors.

## LncRNAs and Tumor Microenvironment

**Figure 2:**
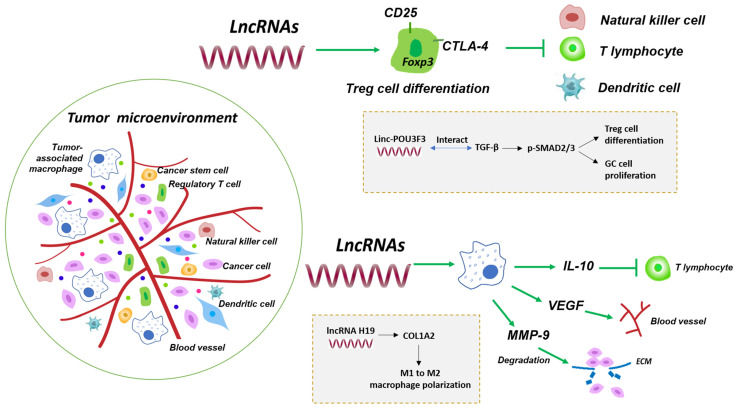
Regulation of lncRNAs in the immune tumor microenvironment. The tumor immune microenvironment consists of not only numerous cancer cells and cancer stem cells, but also a great variety of immune cells, such as regulatory T cells and tumor-associated macrophages. Tumor non-immune microenvironment contains chemokines and cytokines. LncRNAs such as linc-POU3F3 have been shown to interact with intracellular targets and induce T-reg cell differentiation, creating immunosuppressive microenvironment to promote GC cell proliferation 43. Some lncRNAs including lncRNA H19 induce M1 to M2 macrophage polarization that influences release of IL-10, VEGF and MMP-9 and mediates T cell suppression, tumor blood vessel and ECM formation 49.

The hypothesis of “seed and soil” is one of the most influential theories in cancer biology. This theory insists that the initiation and progression of cancer are not only the alterations in tumor cytogenetics and epigenetics, but also influenced by surrounding tumor microenvironment (TME), as the “fertile soil” for the growth of cancerous cells, interacting with each other and coevolving to promote the generations of tumors 30. TME composed of multiple immune cells, stromal cells, extracellular matrix and active mediators in addition to tumor cells, which can be broadly divided into immune microenvironment dominated by immune cells and non-immune microenvironment dominated by fibroblasts and numerous inflammatory factors (Fig. 2) 31. Recently, the advent of tumor immunotherapy, especially targeting immune checkpoints, leads to promising results in the treatments of GC. However, emerging evidence has been reported that heterogeneity of TME is a major hurdle in tumor immunotherapy, which can mediate immunosuppression, leave cancer patients insensitive to immunotherapy, eventually lead to a poor prognosis in the patients with GC. Lifting the immunosuppression of TME is beneficial to the recovery and reconstruction of autoimmunity. Therefore, the therapeutic approaches to TME are promising. Notably, it has become increasingly apparent that lncRNAs have a significant correlation with the TME, which participate in tumorigenicity and progression of GC. Understanding the complex interaction between lncRNAs and TME will eventually excavate prognostic immune markers, which is beneficial to explore both new mechanisms and pathways for blocking tumor immunosuppression, subsequently leading to the stimulation of the host immune response through activating different subsets of immune cells.

The host's own immune response consists of innate immunity, which is represented by macrophages, natural killer (NK) cells and dendritic cells (DC), and specific adaptive immunity, which is dominated by T and B lymphocytes. It seems that immune cells exhibit a bidirectional role in the progression of cancers. Immune cells are responsible for eliminating cancerous cells in the initial stages of tumor invasion, while tumor infiltration lymphocytes (TILs) suppress the effector T lymphocytes activation to help cancer cells immune escape and proliferation during the development of cancer, acting as the accomplice of cancer cells. Among lymphocytes infiltrating in tumor, regulatory T cells (T-regs) and tumor-associated macrophages (TAMs) are the most effective components of the tumor immunosuppressive microenvironment 32.

T-regs are a subset of CD4^+^ T lymphocytes which constitutively expressed interleukin (IL)-2 receptor α-chain (CD25), cytotoxic T lymphocyte antigen-4 (CTLA-4) and the most important lineage differentiation specific factor Foxp3, showing distinct functions of immune impotence and suppression 33. For immune suppression, an elevated expression of IL-2 receptor on T-reg cells could deprive the IL-2 of effectors T cells and inhibit their proliferation 34. On the other hand, the aforementioned another T-reg cell-specific surface molecule, CTLA-4, besides its central role in impeding the activated T cells responses, is involved in T-reg cells-mediated immune suppression 35. Given the indispensable role of T-reg cells on immune suppression, more and more evidence indicated that T-reg cells were implicated in the development of cancers 36-40. Recently, it is recognized that lncRNAs could influence the progression of cancers through regulating the differentiation and functions of T-reg cells (Fig. 2) 41, 42. Xiong *et al.* found that the number of CD4^+^CD25^+^Foxp3^+^ T-reg cells were increased in peripheral blood of GC patients, comparing to the normal samples 43. Further studies indicated that linc-POU3F3 activated the distribution of T-reg and promote the proliferation of GC cells. Mechanically, they observed that linc-POU3F3 could interact with TGF-β which improved the SMAD2/3 phosphorylation level 43. This study showed that the regulation of lncRNAs was capable of promoting the differentiation of T-regs in GC.

Cancer-related inflammation is one of the biological characteristics of tumors 44. Infiltrating macrophages in tissue are derived from bone marrow monocyte precursors, which are mainly divided into M1- and M2-type. Tumor-associated macrophages (TAMs), as the central component of infiltrating leukocytes in tumors, are more similar to the functional phenotype of M2-type macrophages, playing an extremely important role in mediating the occurrence of tumor inflammation and tumor progression 45-48. It is recently found that lncRNA H19 was overexpressed in GC, and was correlated with poor outcomes in patients 49. Meanwhile, lncRNA H19 could regulate the COL1A2 expression level via miRNA-29a-3p. COL1A2 was significantly associated with immune infiltrating cells and lymphatic metastasis, indicating that lncRNA H19 could alter COL1A2 level to enhance the polarization process of macrophages from M1 to M2 in GC, thus promoted the development of GC 49.

**Figure 3:**
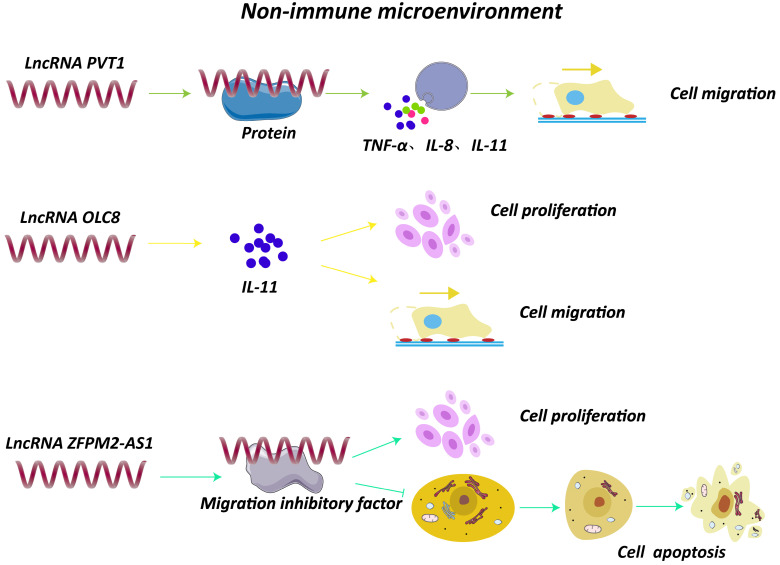
Interaction of lncRNAs with components of tumor non-immune microenvironment. LncRNA PVT1 increased expression of TNF-α, IL-1β and IL-8 and promoted cell migration 9. LncRNA OLC8 bound to IL-11, and the complex significantly suppressed the degradation of IL-11 mRNAs, thus elevating IL-11 expression and promoting cell proliferation and migration 50. LncRNA ZFPM2-AS1 interacted with MIF and prevented degradation of MIF 51.

Besides the immune cells, non-immune TME is composed of multiple extracellular molecules including cytokines and chemokines. Notably, lncRNAs could affect the secretion and synthesis of these small molecule compounds, which may exert a vital role in GC progression (Fig. 3). For example, knockdown of lncRNA PVT1 remarkably decreased the expression of inflammatory factors, such as tumor necrosis factor-α (TNF-α), IL-1β, IL-8, leading to a reduced ability of cell migration 9. A study found that lncRNA H19 induced by *H. pylori* infection was overexpressed in GC cells and tissues, and could activate NF-κB signaling pathway, resulting in an upregulation of pro-inflammatory cytokines and enhancing the progression of GC 7. Moreover, lncRNA OLC8, which was observed highly expressed in GC cell lines and samples, could bind to IL-11, and the complex significantly suppressed the degradation of *IL-11* mRNAs, thus elevated *IL-11* expression and promoted cell proliferation and migration 50. In addition, a novel lncRNA ZFPM2-AS1 was found overexpressed in GC tissues and was able to interact with macrophage migration inhibitory factor (MIF), protecting it from degradation 51. Of note, down-regulation of MIF could attenuate the role of lncRNA ZFPM2-AS1 on p53, revealing that lncRNA ZFPM2-AS1 may participate in GC development through ZFPM2-AS1/MIF/p53 pathway 51.

**Figure 4:**
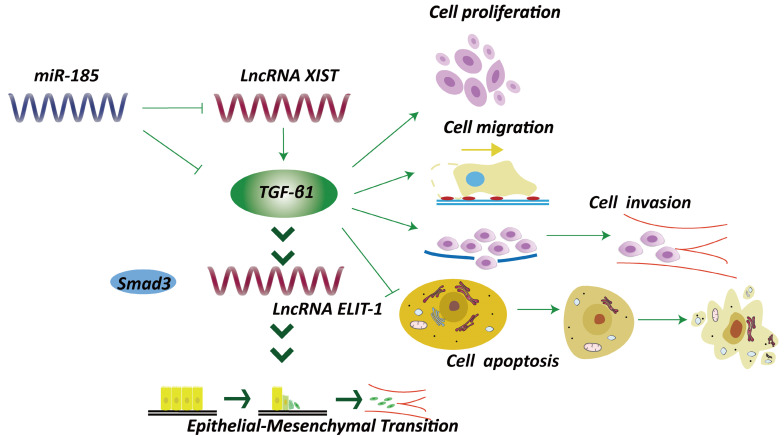
LncRNAs affects TGF-β1 signaling and epithelial-mesenchymal transition. For instance, lncRNA ELIT-1, functioning as Smad cofactor, could be activated by TGF-β1 through the TGF-β1/Smad axis 52. miRNA-185 could inhibit lncRNA XIST and TGF-β1, thus suppressed GC development 53.

TGF-β1 has long been correlated with epithelial-mesenchymal transition (EMT) and tumor progression (Fig. 4). Sakai *et al.* identified a novel lncRNA ELIT-1, which acts as Smad cofactor and could be activated by TGF-β1 through the TGF-β1/Smad axis 52. Knockdown of ELIT-1 inhibited EMT progression mediated by TGF-β1, as well as the expression of downstream target of TGF-β1, including some transcription molecules essential for EMT such as the Snail. Further investigations indicated that lncRNA ELIT-1 directly bound with Smad3, thus positively modulated the target genes activities through recruiting Smad3 to their promoters. The study thus illustrated the roles of lncRNA ELIT-1 in EMT 52. Another lncRNA XIST was overexpressed in GC cells 53. Intriguingly, a negative correlation between lncRNA XIST and miRNA-185 was observed based on bioinformatics analysis, and TGF-β1 was identified as a downstream target of miRNA-185, which could counter-regulate TGF-β1. Knockdown of lncRNA XIST suppressed GC development through elevating miRNA-185 to inhibit TGF-β1. The results revealed that lncRNA XIST/miRNA-185/TGF-β1 pathway was involved in GC development (Fig. 4) 53.

However, the studies on the regulatory role of lncRNAs within GC TME were considered insufficient. The facts that GC is a molecularly and phenotypically highly-heterogeneous disease and has different subtypes with striking distinct clinical features may obstruct the exploration of the accurate and specific lncRNAs which could regulate the T-regs or other components to confer the suppressive activity on tumor immunity. Therefore, to explore the interaction between lncRNA and TME and to find out the exact mechanisms underlying lncRNAs regulate components of TME are among the most urgent and fundamental challenges.

## LncRNAs and Drug Resistance

**Figure 5:**
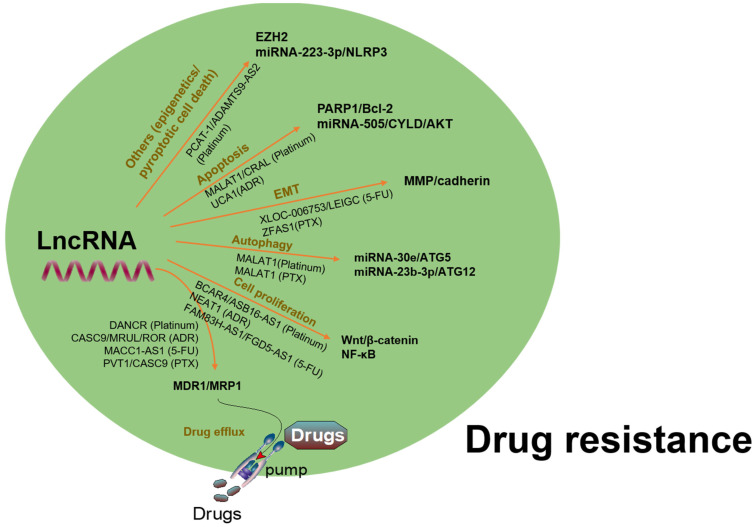
A summary of regulation of lncRNAs in chemoresistance via interaction with different targets and pathways. LncRNAs have been found to be involved in drug resistance in GC by influencing drug efflux, apoptosis, DNA repair, cell cycle, proliferation, autophagy, epithelial-mesenchymal transition, and cancer stem cell through regulating expression of potential target genes and related signaling pathways.

To date, chemotherapy and targeted therapy are commonly used in the treatment of GC. However, the emergence of MDR in GC cells is a major challenge in clinical practice. A variety of lncRNAs have been found to be abnormally expressed in GC and contributed to MDR via regulation of varied target genes and pathways (Fig. 5). Several oncogenic lncRNAs such as PCAT-1, SNHG5, BCAR4, GHET1, HOTAIR, PVT1, MALAT1, UCA1 and NEAT1, as well as certain tumor suppressive lncRNAs, have been demonstrated to involve in MDR of GC (Table 1).

### LncRNAs and Platinum Resistance

Numerous lncRNAs are involved in platinum drugs resistance. For example, Guo *et al.* found that the expression level of lncRNA PCAT-1 was significantly increased in both GC tissues and cells, which contributed to cisplatin drug (DDP) resistance to GC cells 54. Subsequent experiments indicated that lncRNA PCAT-1 could enhance DDP resistance of GC cells via regulating the miRNA-128/ZEB1 axis 54. Another study also demonstrated that knockdown of lncRNA PCAT-1 reversed DDP resistance in GC cells 55. Mechanically, resistance of GC cells to DDP modulated by lncRNA PCAT-1 was attributed to silenced phosphatase and tensin homolog (PTEN) via interacting with enhancer of zeste homolog 2 (EZH2) 55. A recently study demonstrated that lncRNA UCA1 had been correlated with DDP resistance in GC 56. It was found that high expression of lncRNA UCA1 enhanced cell proliferation while suppressed apoptosis induced by DDP, which was related with poor prognosis in GC patients based on TCGA and GEO database. Moreover, lncRNA UCA1 could interact with EZH2 and activate PI3K/AKT signaling pathway, thereby promote the GC cells resistance to DDP 56. Homoplastically, lncRNA DNACR was identified to be overexpressed in GC cells resistant to cisplatin drugs. Knockdown of lncRNA DANCR in BGC823 and SGC7901 GC cells could enhance cell apoptosis as well as suppress cell growth. While overexpressing lncRNA DANCR led to an increase of MDR1, MRP1 expression level which contributed to DDP-resistance in GC cells 57. Yan *et al.* demonstrated that lncRNA HOTAIR, which was remarkably upregulated in DDP-resistant GC cells and tissues, resulting in an enhancement of cancer cell proliferation but an inhibition of cancer cells apoptosis, and accelerated transition of cell cycle G1/S 58. Upregulation of HOTAIR contributed to DDP resistance of GC through interacting with miRNA-126 and decreasing miRNA-126 expression to activate PI3K/AKT/MRP1 signaling pathways 58. Of great interest, overexpression of lncRNA BCAR4 promoted DDP resistance of GC via stimulating the Wnt pathway, while reducing the expression of BCAR4 increased the sensitivity of GC cell to cisplatin 59. It had been reported that lncRNA MALAT1 was emerging player in DDP-resistance of various types of cancers 60-62. A recent study found that GC cells showing cisplatin resistance were often accompanied by an upregulation of lncRNA MALAT1, while using propofol could suppress MALAT1 expression and enhance GC cell apoptosis 63. Mechanically, MALAT1 inhibited autophagy through binding with miRNA-30e, thus regulated ATG5 expression. Collectively, propofol may promote autophagy, thereby enhance the sensitivity of cisplatin drugs in GC through downregulating MALAT1 63. Moreover, lncRNAs HOXD-AS1 64, FAM84B-AS 65, TP73-AS1 66, ASB16-AS1 67, PVT1 68 and HOTTIP 69 also conferred the resistance of GC cells in response to DDP via regulating expression levels of MDR-associated genes.

Despite numerous lncRNAs had been corroborated to be the contributors of DDP-resistance in GC, some lncRNAs function as tumor suppressor and are able to sensitize DDP-resistant GC cells. For example, lncRNA ADAMTS9-AS2 was under-expressed in GC tissues and cells, which could inhibit GC progression by sponging miRNA-223-3p to suppress miRNA-223-3p expression in GC cells 70. In addition, NLRP3 was a downstream target of lncRNA ADAMTS9-AS2 binding with miRNA-223-3p, transposed DDP-resistant GC cells to cisplatin via activating NLRP3 induced pyroptotic cell death 70. Another lncRNA CRAL was identified to be downregulated in cisplatin-resistant GC cells, thereby attenuated cisplatin-induced DNA damage and apoptosis 71. The results indicated that lncRNA CRAL could sponge miRNA-505 to upregulate the cylindromatosis gene (CYLD) expression, which subsequently inhibited AKT activation and resulted in an enhancement in the sensitivity of GC cells to DDP 71.

### LncRNAs and Doxorubicin Resistance

Doxorubicin (DOX) is an antibiotic reagent that is widely used in various cancers, which can inhibit DNA topoisomerase II in tumor cells and induce DNA damage and cell apoptosis. Here, we summarized some lncRNAs that were involved in DOX resistance in GC, including the lncRNAs GAS5, HOTAIR, CASC9, MRUL, UCA1, D63785, HULC, NEAT1 and ROR. Zhang *et al.* found that lncRNA GAS5 expression was downregulated in both GC tissues and cells 72. GAS5 had a lower level in GC cells that were resistant to DOX. Upon restoring GAS5 expression, an inhibition of cell growth while an enhancement in cell apoptosis were observed, suggested that GAS5 functioned as a tumor suppressor and could reverse DOX resistance in GC cells 72. LncRNA CASC9, which was involved in poor differentiation, invasion and metastasis, could promote DOX resistance in GC cells 73. *Shenqi-fuzheng* formula (SQFZ), a Chinese medicine, was able to remarkably boost the sensitivity of DOX to GC cells, and this therapeutic effect was counteracted by the regulation of lncRNA HOTAIR in miRNA-17-5p/PTEN axis. This may indicate that lncRNA HOTAIR was associated with DOX resistance in GC 74.

Moreover, the expression of lncRNA MRUL was remarkably elevated in two chemoresistance GC cells, which could positively affect ABCB1 expression, causing the resistance to DOX in GC 75. Of great interest, lncRNA UCA1 had been found to confer DOX resistance to GC cells via affecting apoptosis-related genes of PARP1 and Bcl-2 76, or through sponging miR-27b 77. Another research found that aberrant expression of lncRNA D63785 could enhance cell growth, migration and invasion of GC cells, and was inversely correlated with microR-422a expression. Further study showed that lncRNA D63785 caused DOX resistance in GC via suppressing miRNA-422-dependent inhibition of the myocyte enhancer factor-2D (MEF2D) 78. In addition, lncRNA NEAT1, which was verified to be overexpressed in GC, could promote cancer cells proliferation and invasion capability 79. Silence of lncRNA NEAT1 in SGC7901 cells reversed the DOX resistance of GC cells 79. LncRNA HULC and ROR were also associated with increased GC cells resistant to DOX 80, 81.

### LncRNAs and 5-Fluoruoracil Resistance

5-Fluoruoracil (5-FU) is a thymidylate synthase inhibitor that affects the synthesis of DNA and proteins. However, cancer cells with 5-FU resistance become a major obstacle to successful chemotherapy for GC patients. There are several oncogenic or tumor suppressive lncRNAs that have been associated with 5-FU resistance, such as FAM83H-AS1, MACC-AS1, HULC, ANRIL, XLOC-006753, FGD5-AS1 and LE1GC. For instance, it was found that the regulation of FAM83H-AS1 in 5-FU resistance was associated with Wnt/β-catenin pathway 82. Multiple lines of evidence indicated that mesenchymal stem cells (MSCs) were related with chemotherapy resistance, but with inconclusive molecular mechanisms. Recently, He *et al.* found that lncRNA MACC-AS1 could be regulated by MSCs, which was able to enhance stemness and 5-FU as well as oxaliplatin resistance through fatty acid oxidation in GC 83. Moreover, overexpression of lncRNAs ANRIL and XLOC-006753 were found to promote 5-FU resistance in GC cells 84, 85. LncRNA XLOC-006753 affected G1/S phase transition, increasing some markers of MDR and EMT expression levels in GC cells 85. In addition, knockdown of lncRNA FGD5-AS1 could inhibit cell growth and sensitivity of GC cells to 5-FU. Further investigations demonstrated that miRNA-153-3p/CITED2 pathway was the downstream target of lncRNA FGD5-AS1 in GC 86. In contrast, tumor suppressor lncRNAs can sensitize the resistance to 5-FU in GC. For instance, overexpression of lncRNA LEIGC was observed to increase the sensitivity of GC cells to 5-FU through suppressing the EMT in GC 87.

### LncRNAs and Paclitaxel Resistance

Paclitaxel (PTX) is one of the first-line chemotherapeutics to handle GC, which results in G_2_/M cell cycle arrest 88. Five oncogenic lncRNAs have been identified to involve in PTX resistance, including the lncRNAs ZFAS1, MALAT1, PVT-1, HOTAIR and CASC9. For instance, lncRNA ZFAS1 contributed to PTX resistance in GC by changing the expression levels of EMT makers, including E-cadherin, N-cadherin, vimentin, matrix metalloproteinase (MMP)-2 and MMP-14, as well as cell cycle related markers (cyclin D1, cyclin E and cyclin B1) 89. Furthermore, it was demonstrated that lncRNA ZFAS1 may stimulate Wnt/β-catenin pathway to enhance PTX resistance in GC 89. LncRNA MALAT1 functioned as a competing endogenous RNA for miRNA-23b-3p and attenuated the suppressive effect of miRNA-23b-3p on ATG12, resulting in PTX chemoresistance in GC cells 90. In addition, lncRNA PVT1was found to overexpress in human GC samples and cells which were resistant to PTX, suggesting that aberrant expression of lncRNA PVT1 may be correlated with chemotherapy efficacy in GC 91. In another study, upregulation of lncRNA HOTAIR contributed to the resistance of PTX in GC. Intriguingly, lncRNA HOTAIR expression had negative correlation with miRNA-217 expression in GC, which indicated that elevated lncRNA HOTAIR may boost PTX resistance in GC cells via suppressing miRNA-217 92. In addition, lncRNA CASC9 was also correlated with PTX resistance of GC through regulating MDR1 protein expression 73.

In conclusion, multiple lncRNAs have been found to be correlated with drug resistance of GC. DDP, DOX, PTX and VCR are all first-line chemotherapeutic reagents to treat GC. However, MDR becomes the primary obstacle of clinical application, which leads to recurrence and poor prognosis. Fortunately, an increasing number of researches have exposed the underlying mechanisms of MDR-related lncRNAs in GC. Treatments towards these abnormally expressed lncRNAs are a promising method to overcome MDR. Furthermore, combination of lncRNAs-based treatment interventions with conventional chemotherapeutics may be a considerable strategy for GC.

## Influences of LncRNAs on Regulating Cellular Signaling Pathways

**Figure 6:**
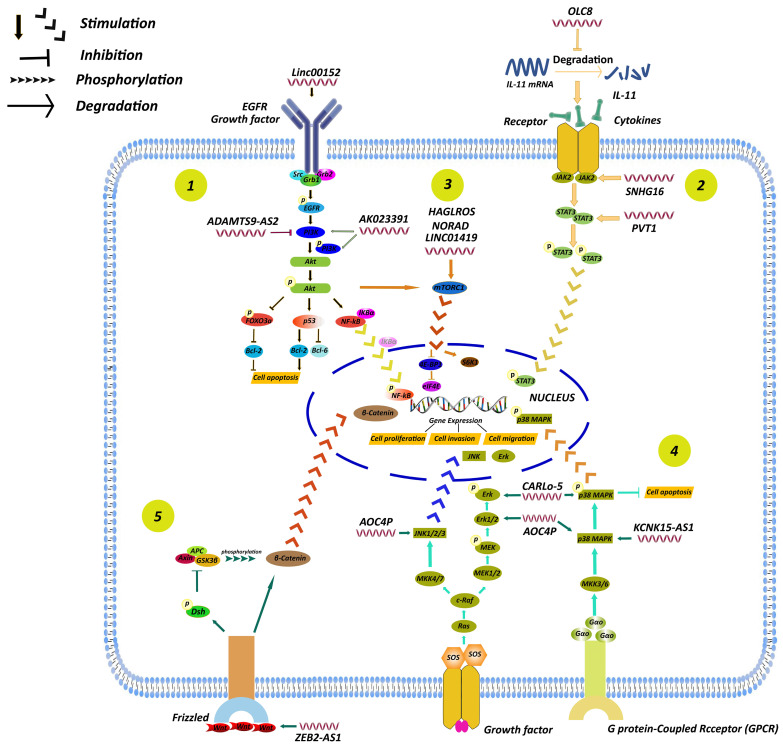
A summary of regulation of lncRNAs in GC through different signaling pathways, including the PI3K/AKT, STAT3, mTOR, MAPK and Wnt/β-catenin signaling pathways. (1) LncRNA AK023391 96 and lncRNA ADAMTS9-AS2 97 participated in GC development by affecting PI3K/AKT signaling pathway. (2) LncRNA PVT1 99, SNHG16 100, and OLC8 50 participated in GC development by affecting STAT3 signaling pathway. (3) LncRNA LINC01419 105, HAGLROS 106, NORAD 107 participated in GC development by affecting mTOR signaling pathway. (4) LncRNA AOC4P 109, CARlo-5 110, KCNK15-AS1 111 participated in GC development by affecting MAPK signaling pathway. (5) Some lncRNAs enhanced GC progression by activating other signaling pathways, including Wnt/β-catenin signaling pathways 112.

Accumulating evidence has indicated that a series of cancer phenotypes attributed to abnormal function of cellular regulator networks. As a given cell signaling pathway may consists of multiple transcription factors, these signaling molecules play a central role in cellular physiological functions and processes. Of great interest, it has become increasingly apparent that lncRNAs are able to participate in the regulation of signaling pathways to affect the development of GC (Fig. 6).

### AKT Signaling Pathway

AKT signaling pathway is a crucial pathway in tumor development, including cell proliferation, migration and invasion, and other biological processes 93. Intriguingly, lncRNAs can influence tumor development by regulating key genes in the AKT pathway. For example, it was demonstrated that linc00152 was closely correlated with cell migration, invasion, apoptosis, cell cycle arrest, and EMT in GC 94. Zhou *et al.* found that down-regulated expression of linc00152 was able to inhibit tumor growth *in vivo* and *in vitro*, and RNA immunoprecipitation (RIP) experiment showed that linc00152 could directly bind to epidermal growth factor receptor (EGFR). Downregulation of linc00152 led to suppressed p-EGFR, p-AKT or p-PI3K, revealing that linc00152 may enhance tumor growth via activation of PI3K-AKT signaling pathway 95.

Recently, a novel lncRNA AK023391 was observed highly expressed in GC tissues comparing with the paired adjust normal tissues, and was closely correlated with lower survival in GC patients 96. Further studies indicated that an elevation of lncRNA AK023391 could promote proliferation, migration and invasion of GC cells 96. Bioinformatics analysis showed that PI3K/AKT and FOXO signaling pathways had the most significant enrichment among the pathways regulated by lncRNA AK023391. It was further confirmed that expression levels of the genes associated with these two mentioned pathways had changed to varying degrees. Downregulation of lncRNA AK023391 repressed tumor growth through PI3K/AKT pathway 96. Therefore, lncRNA AK023391 activated the PI3K/AKT pathway and further affected transcription factors associated with cell proliferation, apoptosis and invasion, resulting in promoting GC tumorigenesis and progression.

On the contrary, lncRNA ADAMTS9-AS2, functioned as a tumor suppressor, was identified to be significantly down-regulated both in GC tissues and cells 97. Cell proliferation, migration and invasion was suppressed in SGC-7901 cells that were transfected with pc-ADAMTS9-AS2 97. Mechanically, p-PI3K and p-AKT expression level was significantly down-regulated in GC cells overexpressed with lncRNA ADAMTS9-AS2. Furthermore, treatment with LY294002 (a PI3K inhibitor) on SGC-7901 cells could reverse the enhanced ability of cell proliferation, cell migration and invasion but a suppression of cell apoptosis when lncRNA ADAMTS9-AS2 expression was down-regulated. These results indicated that the activation of P13K/AKT pathway may participate in the modulation of lncRNA ADAMTS9-AS2 in GC 97.

### STAT3 Signaling Pathway

It is generally known that the signal transducer and activator of transcription 3 (STAT3) pathway participate in a series of biological processes in human cancers, including cell proliferation, survival and apoptosis 98. Vasculogenic mimicry (VM), a vessel-like network which generated by cancer cells with strong invasion ability, exerts a central role in tumor progression. According to Zhao *et al*.'s research, lncRNA PVT1 contributed to VM formation both *in vitro* and* in vivo* 99. Moreover, they observed that lncRNA PVT1 could activate STAT3 and assist STAT3 to recruit Slug promoter, thereby increasing Slug expression and leading to EMT and VM formation 99. Hence, their findings showed that the lncRNA PVT1/STAT3 axis may be a possible therapeutic target in GC 99. Furthermore, lncRNA SNHG16 could act as an oncogene in GC which was attributed to the activation of JAK2/STAT3 signaling pathway via blocking the function of miRNA-135 100. Zhou et al. found that lncRNA OLC8 could interfere with IL-11 mRNA degradation so that the expression level of IL-11 was significantly increased. With no doubt, an elevated expression of IL-11 could enhance STAT3 activation to favor GC development 50.

### mTOR Signaling Pathway

The mammalian target of rapamycin (mTOR) pathway exhibits a pivotal role on cell growth and migration in human cancers, including GC 101, 102. Moreover, mTOR exerts its role through two complexes, mTOR complex 1 (mTORC1) and mTOR complex 2 (mTORC2), which are completely different both in function and structure 103, 104.

Wang *et al.* found that lncRNA LINC01419 was significantly upregulated in GC tissues 105. Downregulation of lncRNA LINC01419 or inhibition of the mTOR pathway, or both, could lead to a suppressive role in migration and invasion of GC cells while promoted cell autophagy 105. AKT1 and mTOR phosphorylation level was decreased upon the inhibition of LINC01419 expression, which may reveal that lncRNA LINC01419 participated in the progression of GC through activating the AKT/mTOR pathway 105.

Autophagy is one of the most essential biological processes with the capable of maintaining cellular homeostasis and protecting against invading pathogens. A study showed that overexpression of lncRNA HAGLROS promoted GC developments and represented poor prognosis via regulating mTOR signaling pathway to interact with mTORC1, which resulted in autophagy suppression 106. Another investigation demonstrated that lncRNA NORAD was overexpressed in GC tissues and cell lines, while miRNA-214 was remarkably down-regulated. LncRNA NORAD accelerated GC cell growth both *in vivo* and *in vitro*. However, an elevated miRNA-214 expression could reserve this effect. Further investigation suggested that lncRNA NORAD may contribute the progression of GC through regulating mTOR signaling pathways 107.

### MAPK Signaling Pathway

Evidences demonstrate that the mitogen-activated protein kinase (MAPK) pathway plays a crucial role in cell growth and consists of key cascades of the ERK1/2, JNK1/2/3, and p38-MAPK 108.

Qu *et al.* found that lncRNA AOC4P was overexpressed in GC tissues, comparing with the paired adjacent tissue, and its high expression was closely related with poor survival in GC patients 109. Knockdown of lncRNA ACO4P led to the inhibition of cell proliferation, migration and invasion, while enhanced the apoptosis in GC cells 109. Expression level of the related proteins, including ERK1, p38 and JNK, were decreased upon the inhibition of lncRNA ACO4P. These data suggested that lncRNA ACO4P participated in GC development via activating the MAPK signaling pathway 109. Similarly, lncRNA CARlo-5 was overexpressed in GC tissues which contributed to tumor growth 110. Knockdown of lncRNA CARlo-5 could inactivate the ERK/MAPK signaling pathway, thereby induced G_0_/G_1_ cycle arrest and apoptosis and dramatically inhibited GC cell proliferation 110. By contrast, overexpression of lncRNA KCNK15-AS1 could inhibit cell growth, while increase cell apoptosis in GC. Subsequent experiment revealed that both DNA methyltransferase (cytosine-5) 1 (DNMT1) and histone deacetylase 1 (HDAC1) were involved in the process of KCNK15-AS1 methylation in GC, and this interaction led to the activation of the MAPK and AKT signaling pathway 111.

### Other Signaling Pathways

Besides the mentioned above signaling pathways, there are other signaling pathways involved in regulation of lncRNAs in GC. For instance, the expression level of lncRNA ZEB2-AS1 was up-regulated in GC cells and clinical samples 112. Knockdown of lncRNA ZEB2-AS1 via infecting ZEB2-AS1-shRNA was capable of inhibiting cell proliferation, metastasis and EMT, while promoting the apoptosis of GC cells 112. Furthermore, western blot experiment indicated that ZEB2-AS1 may contribute to development of GC via modulating the Wnt/β-catenin signaling pathway 112. There was a report revealed that the well-known lncRNA, HOTAIR, was highly expressed in GC cells and tissues via the activation of CXCR_4_ and RhoA signaling pathways 113. Additionally, lncRNA BANCR and ANRIL were both involved in GC progression via regulating NF-κB 114, 115.

## LncRNAs as Biomarkers and Therapeutic Targets in GC

### Diagnostic and Prognostic Role of LncRNAs

Owing to the lack of conspicuous symptoms in early stage, most GC patients are at advanced stage in diagnosis. Hence, it is urgent to identify effective biomarkers for diagnosis. Some established tumor-specific biomarkers, including carcinoembryonic antigen (CEA), carbohydrate antigen (CA) 242, CA724, and serum pepsinogen (SPG), are known to have limitations in the diagnosis of GC 116. LncRNAs can be detected in a highly stable form in plasma and are therefore considered to be promising diagnostic indices. Recently, studies reported that lncRNAs were enriched and stabilized in exosomes that were secreted by cancer cells 117, 118. It was reported that lncRNAs such as ZFAS1, HOTTIP and UUEGC1 could be detected in plasma exosomes and there were distinct differences between GC patients and the controls 119-121. Thus, abundant lncRNAs could be the potential biomarkers in GC diagnosis (Table 2).

Emerging evidence indicated that the levels of lncRNAs in plasma could be satisfactory tumor biomarkers in GC diagnosis. For instance, the expressions of lncRNA H19 in plasma of GC patients were remarkably higher than that in controls, and subsequent ROC analysis showed that the area under the curve (AUC) was 0.838. Furthermore, the expression of H19 distinguished early-stage GC from controls with an AUC of 0.877. These results demonstrated that plasma H19 may be an available detecting biomarker for GC 122.

Beyond that the use of a single lncRNA, the multiple plasma lncRNA combinations could become more powerful for GC diagnosis. Studies suggested that the levels of plasma lncRNAs of FAM49B-AS1, GUSBP11 and CTDHUT in GC patients were significantly higher than that in healthy controls 123. Moreover, the combinational use of these three lncRNAs with CA242 or CA724 was superior to the CA242 or CA724 alone for GC diagnosis 123. Consequently, plasma FAM49B-AS1, GUSBP11 and CTDHUT had great potential as promising tumor biomarkers for early GC diagnosis.

Evaluation of prognosis is essential for assessing treatment status and adjusting treatment strategy. More strikingly, lncRNAs are able to act as prognostic biomarkers in GC (Table 3). For instance, the expression of lncRNA MIR100HG was observed to be overexpressed in clinical samples from GC patients and GC cells, compared with controls or the adjacent normal gastric mucosa tissues. Further study indicated that an elevated level of lncRNA MIR100HG was positively correlated with clinical stage, tumor invasion, lymph node metastasis and distant metastasis. Moreover, survival analysis revealed that MIR100HG expression was negatively related with clinical outcome in patients with GC 124. Therefore, MIR100HG, an oncogenic lncRNA, could be a valuable biomarker in GC prognosis. Similarly, lncRNA Sox2 overlapping transcript (Sox2ot) was highly expressed in GC cell lines and tissues based on Zhang *et al*.'s study 125. The relationship between lncRNA Sox2ot and clinicopathological features was analyzed subsequently. The results showed that the level of Sox2ot was correlated with clinical stage, tumor depth, lymph node metastasis and distant metastasis 125, suggesting that Sox2ot was a potential prognostic marker for GC patients.

### LncRNAs as Therapeutic Targets for GC

Given the fact that lncRNAs are involved in many biological and pathological functions, exerting a crucial role in GC development, progression and metastasis, it is possible to consider exploiting lncRNAs as therapeutic targets in GC. One of advantages of targeting lncRNAs is that they may have pleiotropic effects via influencing multiple tumor-related signaling pathways. As a result, targeting lncRNAs may yield pomissing treatment outcome based on small interference RNAs (siRNAs), antisense oligonucleotides (ASOs), or Crispr-Cas9 techniques.

Some drugs may also target lncRNAs to influence GC growth. Accumulating vigorous evidence indicated that lncRNA PVT1 was overexpressed in a number of cancers including GC 126, and was significantly associated with tumor disease processes 127-130. Cardamonin (CARD, 2′,4′-dihydroxy-6′-methoxychalcone), which is a kind of chalcone, is an active ingredient isolated from several plants such as the Alpinia 131. Wang *et al.* found that CARD was able to inhibit cell proliferation and migration, as well as induce apoptosis in GC cell lines. Furthermore, they observed that CARD remarkably decreased lncRNA PVT1 expression, leading to an inhibition of STAT3 pathway 132. The findings firstly indicated that some drugs may regulate lncRNAs to inhibit GC, and lncRNAs serve as potential targets. Despite that there are few studies on drugs targeting lncRNAs in GC at present, it is clear that there is great potential for developing drugs targeting lncRNAs for the clinical treatment of GC.

## Conclusions and Future Perspectives

GC is one of the most common malignant tumors with high morbidity and mortality worldwide. Emerging evidence indicates that a large number of lncRNAs play a vital role in the maintenance of human homeostasis. Alteration of lncRNAs expression may affect a series of disease process, including cell growth, metastasis, apoptosis, EMT and MDR. Hence, it is essential to clarify the detail mechanisms between lncRNAs and gastric tumorigenesis and developments.

*H. pylori* infection exhibits a pivotal role in GC tumorigenesis, and differentially expressed lncRNAs are associated with *H. pylori*-associated GC. In this review, we summarized the current findings regarding the role of lncRNAs in *H. pylori*-associated GC. Some lncRNAs may be potential diagnostic marker and prognostic factor. Despite the fact that most studies revealed the potential association of lncRNAs in *H. pylori*-induced GC, the relationships between multiple virulence factors considering *H. pylori* infection and the alterations of lncRNAs in gastric diseases, especially GC, are required to illuminate in further investigations.

In decades, it is realized that finding out the key molecules and/or signaling pathways in the TME is essential for future cancer therapy. In this regard, lncRNAs has been found to implicated in regulation of multiple immune cells and cytokines, such as TGF-β superfamily 9, 43, 52, 53, 133, IFN family 134, IL family 9, 50, and related signaling pathways 7, 51, thus regulating the TME, changing the biological functions of tumor, and gradually promoting or inhibiting the progression of GC. However, the studies on the regulatory role of lncRNAs within GC TME were considered insufficient, which may be due to the facts that GC is a molecularly and phenotypically highly-heterogeneous disease and has different subtypes with striking distinct clinical features. Therefore, to explore the interaction between lncRNA and TME and to find out the exact mechanisms underlying the regulation of lncRNAs in TME are among the most urgent and fundamental challenges.

Alteration of lncRNAs were found closely correlated with drug resistance. Nevertheless, the detailed mechanisms may be complicate. The results suggested that knockdown of lncRNAs through small interfering RNAs (siRNAs) or short hairpin RNAs (shRNAs) may benefit to extend understanding of molecular mechanisms underlying MDR of GC as well as the GC treatment. Furthermore, combination of lncRNAs-based therapeutic interventions with traditional chemotherapy seems to be an approach to overcome MDR in GC.

**Figure 7:**
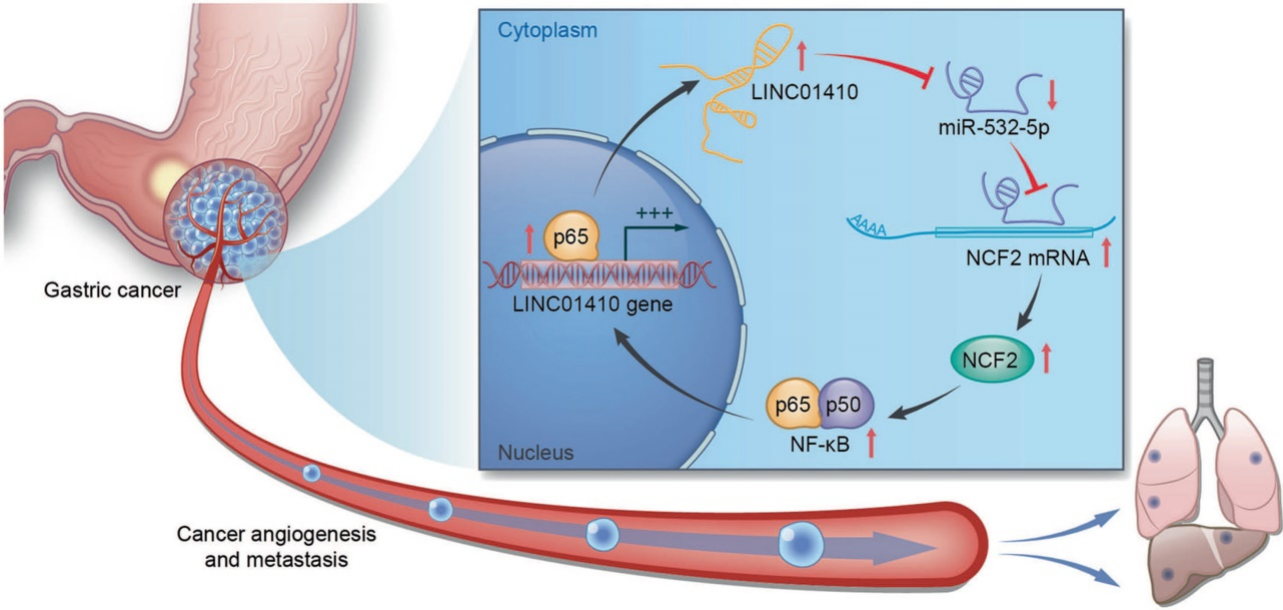
The feedback loop between LINC01410 and NCF2. LINC01410 upregulated NCF2 and NF-κB signal via binding to and inhibiting miRNA-532-5p. NCF2 in turn increased LINC01410 via NF-κB 135. Reprinted with permission 135.

It has become increasingly apparent that lncRNAs are capable of regulating cellular physiological processes and other biological functions by targeting the key genes or regulator through different signaling pathways in GC. Here, we summarized the abnormally expressed of lncRNAs in GC progression as well as the signaling pathway correlated with the tumorigenesis and development of GC. Studies reveal that lncRNAs could become important indexes for GC diagnose and prognosis, which might provide novel therapeutic targets in future GC treatment. Moreover, lncRNAs can also interact with miRNAs that affect gene expression, thereby promoting or suppressing GC progression. For example, miRNA-532-5p was able to suppress GC angiogenesis and metastasis, while linc01410 could bind with miRNA-532-5p which repressed its function. Furthermore, there had been a positive feedback loop between linc01410 and neutrophil cytosolic factor 2 (NCF2) through the NF-κB pathway that promoted GC development (Fig. 7) 135. Thus, this opens novel investigation lines towards the relationship between the dysregulated lncRNAs and GC progression, thereby providing brand new treatment concept.

Many studies have revealed the potential role of lncRNAs as biomarkers in GC. LncRNAs can be detected in a highly stable form in plasma, and testing lncRNAs in plasma is simple, non-invasive and easy to be accepted for GC diagnosis. Either a single lncRNA or the combinations of plasm lncRNAs has been reported to be employed as biomarkers for GC diagnosis. Moreover, lncRNAs can be a credible complement to classic cancer detecting indices. In addition to the use of diagnosis, more and more studies have shown that lncRNAs may act as prognostic factors in GC owing to a closely correlation between the expression levels of lncRNAs and clinical features of GC patients. Furthermore, it is distinct to notice an obvious alteration of levels of lncRNAs after certain clinic treatments. Thus, novel lncRNAs that can be stably used in the diagnosis and prognosis of GC is a promising filed which is needed for further evaluation.

It is as well convincing that lncRNAs can be therapeutic targets in GC, since lncRNAs control tumor micro-regulatory networks, and targeting lncRNAs may affect different tumor-associated pathways 136. Targeting lncRNAs may yield reliable therapeutic results based on small interference RNAs (siRNAs), antisense oligonucleotides (ASOs), Crispr-Cas9 techniques, lncRNA-targeting drugs and so on. However, there are still limitations for translating knowledge on lncRNAs to the clinical practice. First, the current status of mechanism study of lncRNAs in GC is considered inadequate. Second, the vast majority of studies associated with lncRNAs in GC focused on their expression profiles, but the role of lncRNA mutants in GC progression is still a mystery. Nonetheless, there is no doubt about the significance of lncRNAs applications in clinic treatment in future.

Collectively, despite of numerous gaps, recent researches have indicated that a large number of lncRNAs play a crucial role in biological process in GC, including cell growth, metastasis, apoptosis, drug resistance and so on. Studies dedicating to decode the underlying mechanisms of lncRNA-associated GC may provide a rationale in the fight against GC, regarding both biomarkers and therapeutic targets.

## Figures and Tables

**Table 1:** LncRNAs associated with MDR in GC

LncRNAs	Role	Expression	Target genes and/or pathways	Reference
**Platinum resistance**				
PCAT-1	Oncogene	Upregulated	miRNA-128/EZH2	54
UCA1	Oncogene	Upregulated	PI3K/AKT	56
DANCR	Oncogene	Upregulated	MDR1, MRP1	57
HOTAIR	Oncogene	Upregulated	miRNA-126; PI3K/AKT	58
BCAR4	Oncogene	Upregulated	Wnt/β-catenin	59
HOXD-AS1	Oncogene	Upregulated	EZH2	64
FAM84B-AS	Oncogene	Upregulated	Unknown	65
TP73-AS1	Oncogene	Upregulated	HMGB1/RAGE	66
ASB16-AS1	Oncogene	Upregulated	NF-κB	67
HOTTIP	Oncogene	Upregulated	HMGA1/miRNA-218	69
MALAT1	Oncogene	Upregulated	miRNA-30e/ATG5	60-63
PVT1	Oncogene	Upregulated	miRNA-3619-5p/TBL1XR1	68
ADAMTS9-AS2	Tumor suppressor	Downregulated	miRNA-223-3p/NLRP3	70
CRAL	Tumor suppressor	Downregulated	miRNA-505/CYLD	71
**DOX resistance**				
GAS5	Tumor suppressor	Downregulated	Unknown	72
HOTAIR	Oncogene	Upregulated	miRNA-17-5p	74
CASC9	Oncogene	Upregulated	MDR1	73
MRUL	Oncogene	Upregulated	ABCB1	75
UCA1	Oncogene	Upregulated	PARP1, Bcl-2, miRNA-27b	76, 134
D63785	Oncogene	Upregulated	miRNA-422a	78
NEAT1	Oncogene	Upregulated	Unknown	79
ROR	Oncogene	Upregulated	MRP1	81
**5-FU resistance**				
FAM83H-AS1	Oncogene	Upregulated	miRNA-145-5p	82
MACC1-AS1	Oncogene	Upregulated	MDR1, MRP1	83
ANRIL	Oncogene	Upregulated	PI3K/AKT/mTOR	84
XLOC_006753	Oncogene	Upregulated	miRNA-153-5p/CITED2	85
FGD5-AS1	Oncogene	Upregulated	Unknown	86
LEIGC	Tumor suppressor	Downregulated	miRNA-145-5p	87
**Paclitaxel resistance**				
ZFAS1	Oncogene	Upregulated	Wnt/β-catenin	89
MALAT1	Oncogene	Upregulated	miRNA-23b-3p	90
PVT1	Oncogene	Upregulated	MDR1, MRP, mTOR and HIF-1α	91
HOTAIR	Oncogene	Upregulated	miRNA-217	92
CASC9	Oncogene	Upregulated	MDR1	73

**Table 2:** LncRNAs as diagnostic biomarkers in GC

LncRNAs	Expression	Diagnostic accuracy	Reference
H19	Upregulated	AUC, 0.838; sensitivity, 82.9%; specificity, 72.9%	122
FAM49B-AS, GUSBP11, CTDHUT	Upregulated	AUC, 0.818	123
GNAQ-6:1	Downregulated	AUC, 0.732	137
B3GALT5-AS1	Upregulated	AUC, 0.816	138
UCA1	Upregulated	AUC, 0.759; sensitivity, 66.67%; specificity, 87.04%	139
UEGC1	Upregulated	AUC, 0.8406	121
PANDAR, FOXD2-AS1, SMARCC2	Upregulated	AUC, 0.839	140
TINCR, CCAT2, AOC4P, BANCR, LINC00857	Upregulated	AUC, 0.91	141

Note: AUC, area under the curve of ROC analysis.

**Table 3:** LncRNAs as prognostic biomarkers in GC

LncRNAs	Expression	Prognostic implications	Reference
SNHG6	Upregulated	Correlated with invasion depth, lymph node metastasis, and distant metastasis	142
SNHG8	Upregulated	Predicted poor prognosis and shorter survival time	143
Sox2ot	Upregulated	Correlated with TNM stage, tumor depth, lymph node metastasis, and distant metastasis	125
MALAT1	Upregulated	Correlated with distant metastasis	144
NEAT1	Upregulated	Correlated with clinical stage, histological type, lymph node metastasis, and distant metastasis	145
MIR100HG	Upregulated	Correlated with TNM stage, tumor invasion, lymph node metastasis, and distant metastasis	124
CTD-2510F5.4	-	Correlated with pathological grade, vascular or nerve invasion, TNM stage and OS, shorter MST	146

Note: TNM, Tumor node metastasis; OS, Overall survival; MST, Median survival time.

## Abbreviations

DOX: Doxorubicin
CagA: Cytotoxin associated gene A
DDP: Cisplatin drug
DSBs: Double-strand breaks
EMT: Epithelial-mesenchymal transition
ERK: Extracellular signal-related kinases
GC: Gastric cancer
*H. pylori*: *Helicobacter pylori*
HR: Homologous recombination
IL: Interleukin
JNK: Jun amino terminal kinases
MAPK: Mitogen-activated protein kinase
MDR: Multi-drug resistance
MIF: Macrophage migration inhibitory factor
miRNA: MicroRNA
MMP: Matrix metalloproteinase
MSCs: Mesenchymal stem cells
mTOR: Mammalian target of rapamycin
ncRNA: Non-coding RNA
NHEJ: Nonhomologous end joining
PTX: Paclitaxel
STAT3: Signal transducer and activator of transcription 3
TAMs: Tumor-associated macrophages
TILs: Tumor infiltration lymphocytes
TME: Tumor microenvironment
T-reg: Regulatory T cell
VacA: Vacuolating cytotoxin A
5-FU: 5-Fluoruoracil
TNM: Tumor node metastasis
OS: Overall survival
MST: Median survival time
